# Causal relationships between gut microbiota, immune cell, and Henoch-Schönlein Purpura: a two-step, two-sample Mendelian randomization study

**DOI:** 10.3389/fimmu.2024.1450544

**Published:** 2024-08-14

**Authors:** Tian Liang, Huijun Shi, Han Cui, Yaqi Cui, Ziwei Zhao, Yue Wang, Dandan Shi, Peichao Tian

**Affiliations:** Department of Pediatric Neurology, First Affiliated Hospital of Zhengzhou University, Zhengzhou, China

**Keywords:** HSP, gut microbiota, immune cell, Mendelian randomization, genetic approaches

## Abstract

**Background:**

Regulating the immune system is a crucial measure of gut microbiota (GM) that influences the development of diseases. The causal role of GM on Henoch-Schönlein Purpura (HSP) and whether it can be mediated by immune cells is still unknown.

**Methods:**

We performed a two-sample Mendelian randomization study using an inverse variance weighted (IVW) method to examine the causal role of GM on HSP and the mediation effect of immune cells between the association of GM and HSP.

**Results:**

We demonstrated the causal relationships between 14 axas and 6 pathways with HSP. Additionally, we identified 9 immune cell characteristics associated with HSP. Importantly, through mediation MR analysis, we identified several immune cell characteristics that mediate the impact of GM on HSP. For instance, Genus_Blautia affects HSP via Monocyte (HLA DR on CD14+ CD16- monocyte) and Monocyte (HLA DR on monocyte). The proportion of mediation effects further elucidated the complex dynamics between GM exposure, immune markers, and their combined impact on HSP.

**Conclusion:**

The study suggested a causal relationship between GM and HSP, which may be mediated by immune cells.

## Introduction

Henoch-Schönlein Purpura (HSP) is an acute vasculitis that predominantly affects children. The exact etiology of HSP remains unclear, though it is believed to be triggered by various factors, including infections and vaccinations. The disease primarily involves inflammation of small blood vessels, affecting multiple organs such as the skin, joints, gastrointestinal system, and kidneys. Symptoms vary depending on the organs involved. Laboratory tests have limited utility in diagnosing HSP, with diagnosis mainly relying on clinical manifestations such as purpura on the skin, arthritis, abdominal pain, and signs of renal impairment. In most cases, HSP is a self-limiting disease. However, severe gastrointestinal complications such as intestinal bleeding or perforation may necessitate hospitalization and surgical intervention to manage these complications (1).

With the increasing prevalence of autoimmune and immune-mediated diseases, research on the relationship between gut microbiota and autoimmune conditions has been intensifying. An accumulating body of evidence suggests that dysbiosis of the gut microbiota contributes to immune pathogenesis, potentially having a causal relationship with the onset or exacerbation of immune-related diseases (2). Microorganisms accompany humans from birth, forming a mutualistic relationship with their host and serving as the primary source of health-influencing microbes. The gut microbiota, which constitutes two-thirds of the human microbial community, is particularly crucial. It maintains gastrointestinal homeostasis and is vital for various functions, including nutrition, metabolism, detoxification, vitamin synthesis, and immune homeostasis (3). Increasingly, scholars believe that the gut microbiota plays a crucial role in regulating the body’s immune responses. Studies using 16S rRNA gene-based pyrosequencing have found that children with HSP exhibit lower gut microbiota diversity and richness compared to healthy children (4). Additionally, *in vitro* animal experiments have shown that mucosal immunoglobulin A (IgA) can specifically target certain bacterial groups in the gut, thereby influencing the abundance of these specific bacterial populations (5).

Circulating immune cells and their subsets are critical components of the human immune system. The pathogenesis of HSP remains to be fully elucidated; however, multiple studies suggest that pathogen infection and the deposition of circulating immune complexes are closely associated with the onset and prognosis of HSP, involving circulating immune cells in these processes (1, 6). Current evidence indicates that both cellular and humoral immunity play roles in the pathogenesis of HSP. A decrease in cellular immunity leads to increased production of inflammatory mediators and heightened secretion of immunoglobulins, which in turn mediate systemic small vessel vasculitis (7). Regulating inflammation and immune responses is a key mechanism by which gut microbiota (GM) influence disease occurrence and progression. GM can alter the local gut environment in various ways, such as by specifically binding to intestinal immunoglobulins and improving the gut barrier function, thereby helping to prevent pathogens from penetrating the intestinal mucosal layer (8, 9). To date, an increasing body of evidence suggests a correlation between HSP, GM, and immune cells. However, most studies have focused on patients in the acute phase of HSP, and the findings may be influenced by potential biases due to reverse causality and unmeasurable confounding factors. Consequently, the causal relationship among these three elements remains unclear.

In recent years, numerous studies have explored the relationship between genetic statistics and immune prospects, revealing how genetic variations influence an individual’s immune response and disease progression. For example, investigations into how genetic variations within tumors affect the tumor-host immune environment have uncovered significant associations between specific genetic abnormalities and immune responses (10). Additionally, research analyzing the spatiotemporal dynamics of immune cells within tumors has demonstrated how different genetic diversities influence the formation of the tumor immune environment (11). MR analysis can delve into the causal relationships between diseases and immune responses, helping to identify potential therapeutic targets and enhancing our understanding of immune-related diseases. Numerous studies have already identified relationships between circulating immune cells and various diseases, such as hypertension, hepatocellular carcinoma, and diabetic nephropathy (12–14).

Mendelian Randomization (MR) analysis is an increasingly prominent method that provides novel perspectives and deep insights into the etiology of diseases by using genetic variation as instrumental variables. This approach not only aids in identifying potential therapeutic interventions but also effectively reduces the confounding and reverse causality issues inherent in traditional observational studies when exploring the roles of complex factors such as gut microbiota and immune cells in disease pathogenesis. MR analysis estimates the causal relationships between exposure factors and diseases or other outcomes through unbiased instrumental variables, thereby minimizing the influence of confounding factors (15).

Recent advancements in two-step MR methods have further enhanced the precision of this analysis, demonstrating less bias compared to multivariable methods when detecting mediators in causal pathways. Previous MR studies have already revealed causal links between immune cell subtype counts and various diseases, such as asthma and rheumatoid arthritis (RA), providing important clues for understanding disease susceptibility. Overall, MR analysis offers a robust statistical tool for etiological research, helping to uncover underlying disease mechanisms and providing scientific evidence for future therapeutic strategies (16).

In this study, we employed a two-step MR approach to determine: (i) whether gut microbiota is causally associated with HSP; (ii) whether immune cells are causally associated with HSP; and (iii) to assess the extent to which immune cells mediate the effects of gut microbiota on HSP.

## Methods

### Study design

The MR analysis follows three fundamental principles: (I) the genetic variation is directly related to the exposure; (II) the genetic variation is not related to potential confounding factors between the exposure and the outcome; and (III) the genetic variation does not affect the outcome through pathways other than the exposure (15).

**Figure 1:**
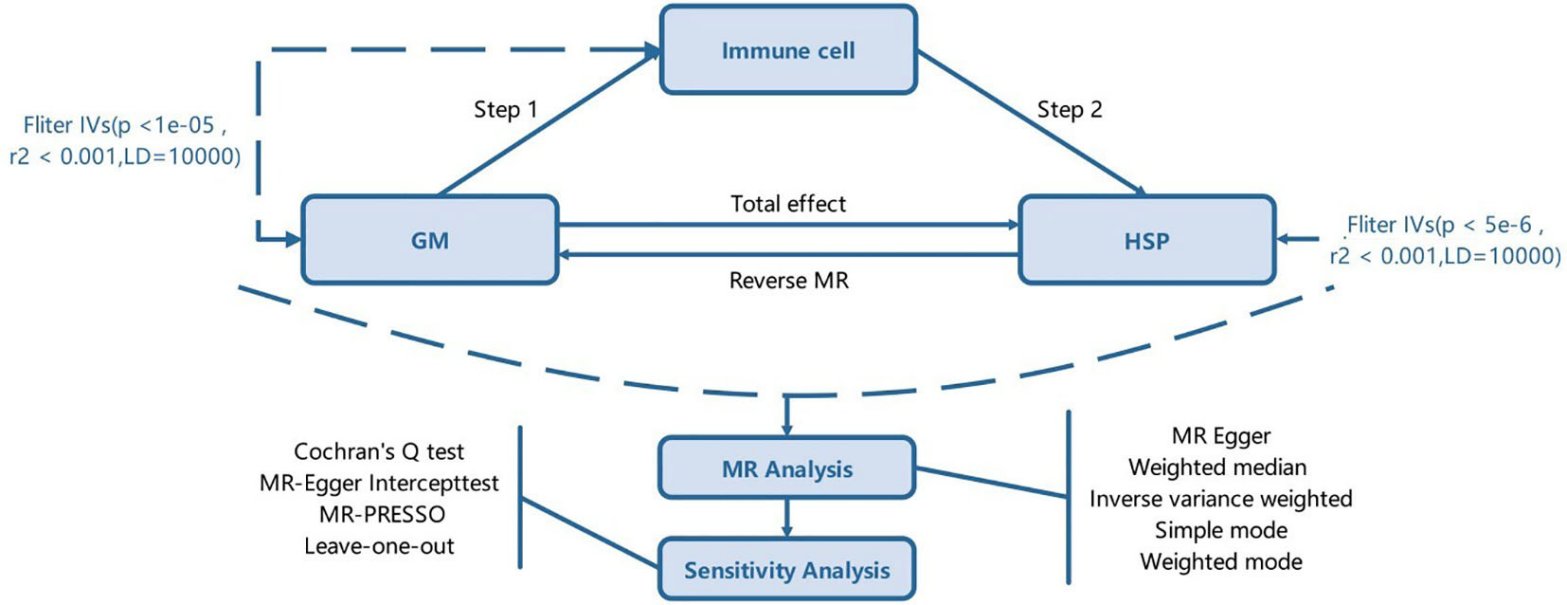
The study design. A two-step Mendelian randomization study of GM on HSP mediated by immune cell. GM, Gut microbiota; HSP, Henoch-Schönlein Purpurar; IVs, Instrumental variables.

The experimental design is illustrated in **Figure 1**. The study is divided into two stages. In the first stage, we used a two-sample MR method, considering GM and immune cell traits as exposure factors and HSP as the outcome. We aimed to identify GM and immune cell traits highly associated with HSP risk. Additionally, we conducted a reverse MR analysis using SNPs associated with HSP as instrumental variables (IVs) to explore potential causal relationships between HSP and the identified gut microbiota traits. GM traits that were positive in the reverse MR analysis were excluded from further analysis. In the second stage, after identifying GM and immune cell traits related to HSP risk, we further assessed the causal effects of GM on these immune cell traits and calculated the proportion of the effect of GM on HSP mediated by each immune cell trait. Through this rigorous approach, we aim to elucidate the causal relationship between GM and HSP and explore the potential role of immune cell traits in this process.

### GWAS summary data sources

Publicly available HSP data comes from the Finn-b-R10_D3_ALLERGPURPURA dataset, which includes 939 HSP cases and 405,762 control cases. The Dutch Microbiome Project provides gut microbiome data from a subset of 8,208 volunteers from the Dutch Lifeline population cohort. From this study, we collected summary statistics for the GM, including 207 taxa and 205 pathways (17). GWAS summary statistics for each immune signature are publicly available from the GWAS Catalog (accession numbers GCST90001391 to GCST90002121) (18). These data encompass 731 immunophenotypes, including absolute cell (AC) counts (n=118), median fluorescence intensity (MFI) reflecting surface antigen levels (n=389), morphological parameters (MP) (n=32), and relative cell (RC) counts (n=192). Specifically, the MFI, AC, and RC features cover B cells, dendritic cells (CDC), mature T cells, monocytes, myeloid cells, TBNK (which includes T cells, B cells, and natural killer cells), and regulatory T cells (Treg). The MP features include CDC and TBNK groups (19). The initial GWAS was conducted using data from 3,757 European individuals, with no overlapping cohorts, identifying approximately 17.6 million genetic variants. Among these, 22% had not been discovered in previous sequencing studies and were enriched for predicted functional consequences. Using a Sardinian sequence-based reference panel, around 22 million SNPs were imputed from high-density array genotyping. The associations were assessed while considering covariates, including sex, age, and age squared (18). All data for GWAS is sourced from different alliances or agencies, so there is no duplication between samples. The definitions of exposure and outcome are detailed in the original article(**Supplementary Table S1**).

### Ethics statement

Genome-wide association studies aggregate statistical data on HSP, microbiota, and immune cells for MR analysis. Every GWAS participating in this research was disclosed via the initial study and obtained ethical clearance from their individual institutions.

### Instrumental variable selection and data harmonization

Due to the random assignment of genetic variation during intergenerational transmission, similar to the random grouping in randomized controlled trials, it has the advantages of reducing confounding factors and enhancing the robustness and reliability of causal inference. Therefore, we gathered single nucleotide polymorphism (SNP) data to serve as IVs for exposure and outcome (20, 21), employing rigorous criteria for their selection. Drawing on previous relevant studies (22), we set the filtering conditions for SNPs to act as IVs for GMs and immune cell traits at a p-value threshold of less than 1e-5, However, when HSP was considered as the exposure, We found that even with a stricter threshold of 5e-6, we could still obtain a sufficient number of instrumental variables for further analysis. Therefore, we increased the threshold to ensure the feasibility supported by an adequate number of instrumental variables, further ensuring the accuracy and reliability of the analysis. To maintain the independence of the selected IVs and mitigate the impact of linkage disequilibrium, we utilized the two-sample MR R package for cluster analysis, setting a threshold of R2 < 0.001 and a cluster distance of 10,000 kb (22, 23). To mitigate biases arising from weak instrumental variables, we specifically calculated the F-statistic, with IVs having an F-statistic exceeding 10 deemed to possess adequate statistical strength. All SNPs that passed the aforementioned selection criteria were included in the final analysis to ensure the accuracy and effectiveness of their causal relationships with GM, immune cell traits, and the risk of HSP (14). The final set of all IVs is displayed in **Supplementary Table S2**.

### Statistical analysis

This study utilized R statistical software version 4.3.2 and the packages “TwoSampleMR,” “VariantAnnotation,” and “ieugwasr” for two-sample MR analysis, aiming to explore the causal relationships between specific exposure factors and outcomes (24). Five MR analysis methods were employed: MR Egger, Weighted median, Inverse variance weighted (IVW), Simple mode, and Weighted mode. Among these, the IVW method was considered the primary approach for assessing causality due to its precision and robustness. Statistical significance was determined with a p-value threshold of less than 0.05, and the odds ratio (OR) was used to measure the association between exposure factors and outcomes. An OR greater than 1 indicated a positive association, while an OR less than 1 indicated a negative association (25, 26).

During the analysis, Cochran’s Q statistic was used to assess heterogeneity. In cases of significant heterogeneity, MR-Egger regression was applied to analyze potential pleiotropy. Pleiotropy was further assessed using the MR-Egger intercept test and the MR-PRESSO method. Leave-one-out sensitivity analysis was performed to evaluate the impact of individual SNPs on the overall causal effect. Additionally, funnel plots and scatter plots were employed to visually present potential pleiotropy (27). Given the multiple datasets processed and compared simultaneously, there was a risk of false-positive results due to random effects. Therefore, the false discovery rate (FDR) method was used for correction, ensuring that only results with p-values less than the FDR threshold were included (28). This comprehensive approach aimed to minimize bias and provide reliable estimates of causal relationships between exposures and outcomes.

## Results

### Total effect of GM on HSP

**Figure 2:**
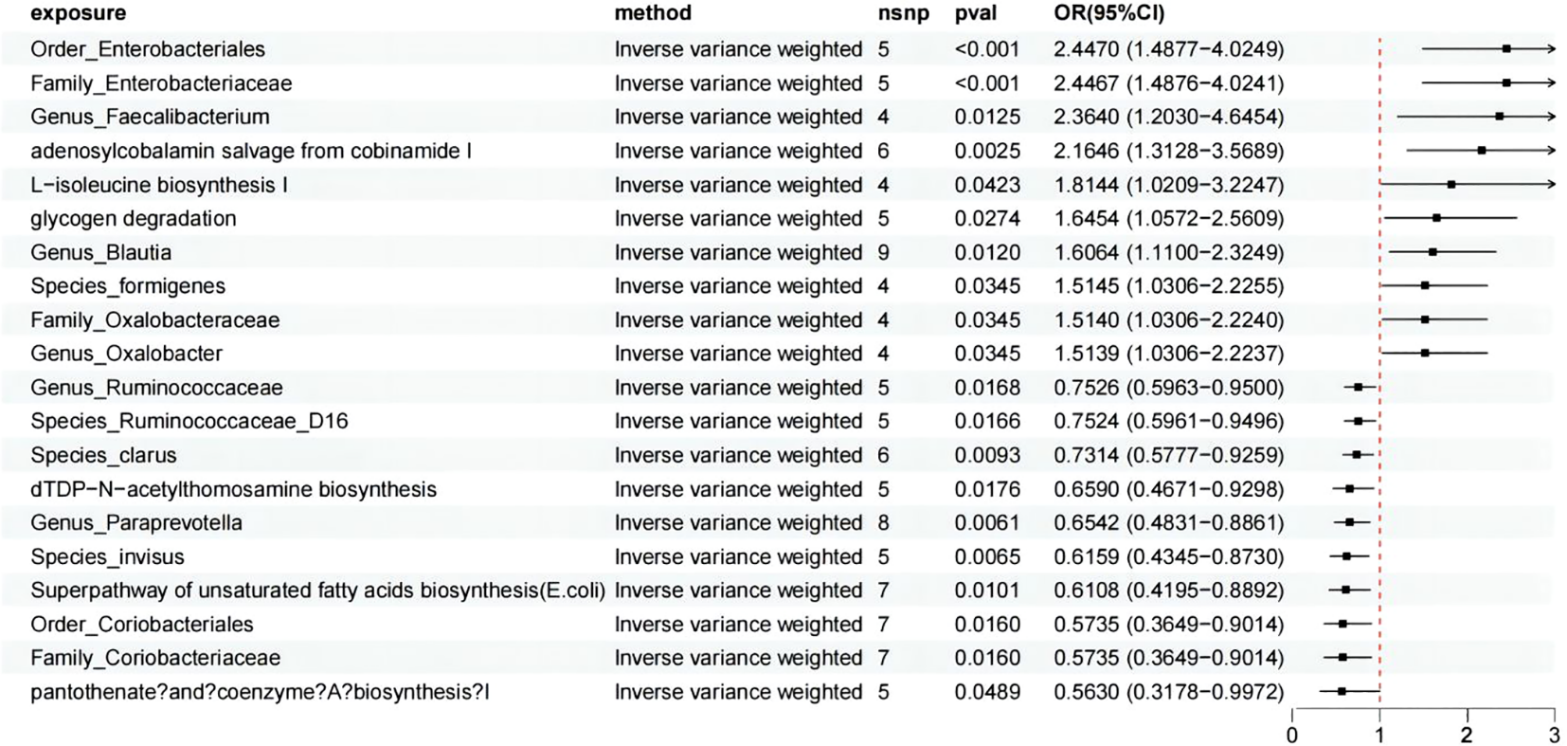
MR analysis showed the causality of 14 axes and 6 pathways on HSP were significant. CI, Confidence Interval; MR, Mendelian randomization; OR, odds ratio.

Through a two-sample MR analysis, we demonstrated a causal relationship between 14 axes and 6 pathways and HSP (**Figure 2**). Among these, 3 pathways and 7 axes showed a positive correlation with HSP, which are as follows: adenosylcobalamin salvage from cobinamide I (OR = 2.165, 95% CI: 1.313-3.569, p = 0.002), glycogen degradation I (OR = 1.645, 95% CI: 1.057-2.561, p=0.027), L-isoleucine biosynthesis I (from threonine) (OR = 1.814, 95% CI: 1.021-3.225, p = 0.042), Family_Oxalobacteraceae (OR = 1.514, 95% CI: 1.031-2.224, p = 0.035), Family_Enterobacteriaceae (OR = 2.447, 95% CI: 1.488-4.024, p = 0.000), Genus_Blautia (OR = 1.606, 95% CI: 1.110-2.325, p = 0.012), Genus_Faecalibacterium (OR = 2.364, 95% CI: 1.203-4.645, p = 0.012), Genus_Oxalobacter (OR = 1.514, 95% CI: 1.031-2.224, p = 0.035), Order_Enterobacteriales (OR = 2.447, 95% CI: 1.488-4.025, p = 0.000), Species_formigenes (OR = 1.514, 95% CI: 1.031-2.225, p = 0.035).

On the other hand, 3 pathways and 7 axes exhibited a protective effect against HSP, which are as follows: pantothenate and coenzyme A biosynthesis I (OR = 0.563, 95% CI: 0.318-0.997, p = 0.049), Superpathway of unsaturated fatty acids biosynthesis (E. coli) (OR = 0.611, 95% CI: 0.420-0.889, p = 0.010), dTDP-N-acetylthomosamine biosynthesis (OR = 0.659, 95% CI: 0.467-0.930, p = 0.018), Family_Coriobacteriaceae (OR = 0.574, 95% CI: 0.365-0.901, p = 0.016), Genus_Paraprevotella (OR = 0.654, 95% CI: 0.483-0.886, p = 0.006), Genus_Ruminococcaceae (OR = 0.753, 95% CI: 0.596-0.950, p = 0.017), Order_Coriobacteriales (OR = 0.574, 95% CI: 0.365-0.901, p = 0.016), Species_Ruminococcaceae_D16 (OR = 0.752, 95% CI: 0.596-0.950, p = 0.017), Species_invisus (OR = 0.616, 95% CI: 0.434-0.873, p = 0.006), Species_clarus (OR = 0.731, 95% CI: 0.578-0.926, p = 0.009). For more detailed information, we present it in **Supplementary Table S3**. As for the picked GMr, we conducted a reverse MR and did not detect any significant causal relationship among them.

In this process, although not all analytical methods yielded statistically significant results, we ensured the consistency in the direction of results across five different analytical methods. Cochran’s Q statistic, MR-Egger intercept test, and MR-PRESSO indicated no heterogeneity or horizontal pleiotropy in this MR analysis (**Supplementary Table S5**). Furthermore, in the leave-one-out sensitivity analysis, no single SNP significantly violated the overall effect of GM on HSP. After FDR correction, the aforementioned results remained significant. Following Bonferroni correction, adenosylcobalamin salvage from cobinamide I (p = 0.049), Family_Enterobacteriaceae (p = 0.008), and Order_Enterobacteriales (p = 0.008) continued to exhibit significance.

### Effect of immune cell traits on HSP

**Figure 3:**
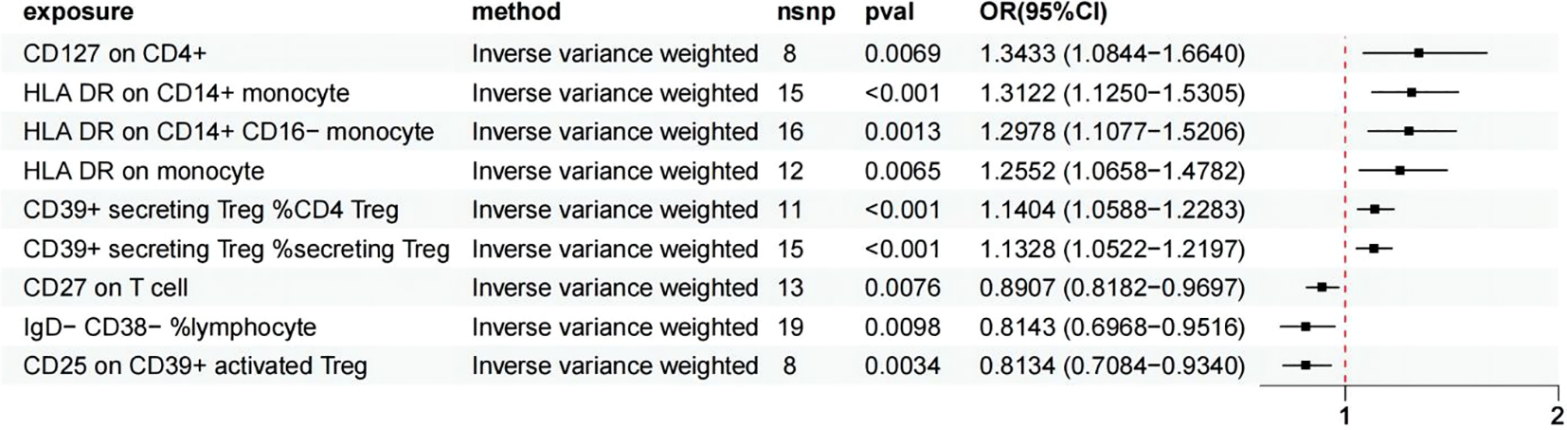
MR analysis showed 6 immune cell traits had protective effects on HSP and 3 immune cell traits had disadvantageous effects on HSP.

Using the IVW method as the primary evaluation standard, we detected that three immune cell traits had a protective effect against HSP: B cell (IgD- CD38- %lymphocyte) (OR = 0.814, 95% CI: 0.697-0.952, p = 0.0100), B cell (CD27 on T cell) (OR = 0.891, 95% CI: 0.818-0.970, p = 0.0076), and Treg (CD25 on CD39+ activated Treg) (OR = 0.813, 95% CI: 0.708-0.934, p = 0.0034). Treg (CD39+ secreting Treg %secreting Treg) (OR = 1.133, 95% CI: 1.052-1.220, p = 0.0009), Treg (CD39+ secreting Treg %CD4 Treg) (OR = 1.140, 95% CI: 1.059-1.228, p = 0.0005), Treg (CD127 on CD4+) (OR = 1.343, 95% CI: 1.084-1.664, p = 0.0069), Monocyte (HLA DR on CD14+ CD16- monocyte) (OR = 1.298, 95% CI: 1.108-1.521, p = 0.0013), Monocyte (HLA DR on CD14+ monocyte) (OR = 1.312, 95% CI: 1.125-1.531, p = 0.0005), and Monocyte (HLA DR on monocyte) (OR = 1.255, 95% CI: 1.066-1.478, p = 0.0065) were six immune cell phenotypes found to increase the risk of HSP (**Figure 3**), More detailed information is presented in **Supplementary Table S3**.

In this MR study, the p-values obtained from Cochran’s Q test were greater than 0.05, indicating no significant heterogeneity. Additionally, both the MR-Egger intercept test and the MR-PRESSO analysis provided no evidence of horizontal pleiotropy (**Supplementary Table S5**). Further leave-one-out sensitivity analyses confirmed the robustness of the causal estimates, as the overall causal relationship estimate remained largely unchanged even when any single SNP was excluded. After FDR correction, the results remained significant. Following Bonferroni correction, Treg (CD39+ secreting Treg %secreting Treg) (p = 0.008), Treg (CD39+ secreting Treg %CD4 Treg) (p = 0.004), Treg (CD25 on CD39+ activated Treg) (p = 0.031), Monocyte (HLA DR on CD14+ CD16- monocyte) (p = 0.011), and Monocyte (HLA DR on CD14+ monocyte) (p = 0.005) continued to show significance.

### Effect of GM on immune cell traits

**Figure 4:**
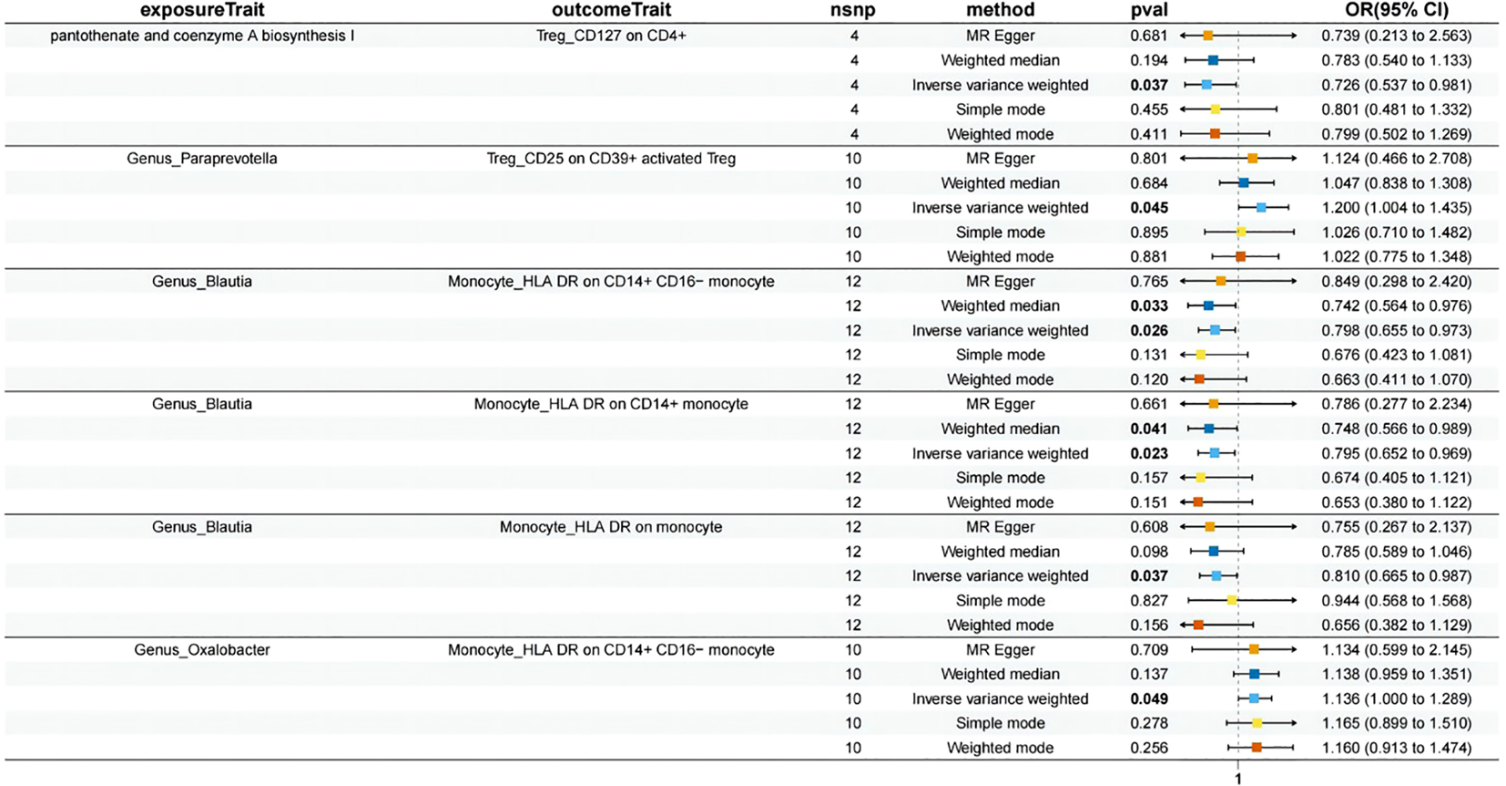
Mendelian randomization analysis between GM and Mediator.

We selected 20 GMs as exposure factors and 9 immune cell types as outcomes for the MR analysis, which resulted in 6 positive associations, as shown in **Figure 3** and **Supplementary Table S4**. Additionally, we performed mediation analysis to investigate the mediating effects of the immune cells in these associations. The specific results are presented in **Table 1** and **Figure 4**.

**Table 1:** Mediation effect of GM on HSP via immune cell.

Traits of GM	Traits of Immune cell	Total effect	Direct effect A	Direct effect B	Mediation effect	Mediation Proportion (%)
		β (95% CI)	β (95% CI)	β (95% CI)	β (95% CI)	
pantothenate and coenzyme A biosynthesis I	Treg (CD127 on CD4+)	−0.5727 (−1.1421−−0.0027)	−0.3183 (−0.6203−−0.0189)	0.2955 (0.0784- 0.5092)	-0.00343 (-0.084, 0.0771)	16.5%
Genus_Paraprevotella	Treg (CD25 on CD39+ activated Treg)	−0.4201 (−0.7263−−0.1211)	0.1823 (0.0037−0.3615)	−0.2059 (−0.3488-−0.0708)	-0.0377 (-0.081, 0.00566)	8.88%
Genus_Blautia	Monocyte (HLA DR on CD14+ CD16- monocyte)	0.4700 (0.1038−0.8396)	−0.2245 (−0.4201−−0.0274)	0.2636 (0.1024- 0.4192)	-0.0587 (-0.119, 0.00207)	-12.4%
Genus_Blautia	Monocyte (HLA DR on CD14+ monocyte)	0.4700 (0.1038−0.8396)	−0.2300 (−0.4283−−0.0321)	0.2728 (0.1187-0.4252)	-0.0625 (-0.124, -0.000608)	-13.2%
Genus_Blautia	Monocyte (HLA DR on monocyte)	0.4700 (0.1038−0.8396)	−0.2107 −0.4078−−0.0130)	0.2276 (0.0649-0.3864)	-0.0479 (-0.104, 0.00793)	-10.1%
Genus_Oxalobacter	Monocyte (HLA DR on CD14+ CD16- monocyte)	0.4149 (0.0295-0.7976)	0.1274 (0.0004-0.2510)	0.2636 (0.1024- 0.4192)	0.0331 (-0.0112, 0.0775)	7.99%

Total effect: The causal role of GM on HSP.

Direct effect A: The causal role of GM on immune cell traits.

Direct effect B: The causal role of immune cell traits on HSP.

β (indirect effect) = β (Direct effect A) * β (Direct effect B).

The mediated proportion = β (indirect effect)/β (total effect).

## Discussion

In recent years, the close relationship between GM and host health has increasingly become a focus of medical research. HSP, a common small vessel vasculitis, has garnered particular attention for its potential association with GM. HSP manifests in various clinical symptoms, including skin purpura, gastrointestinal issues, joint pain, and renal involvement. Multiple studies suggest that GM imbalance or dysregulation may play a critical role in the pathogenesis of HSP.

GM dysregulation may be related to the pathogenesis and clinical manifestations of HSP. An observational study analyzing 18 primary cases, 16 recurrent cases, and 23 healthy children found that the diversity and richness of GM in HSP patients were significantly reduced, and the structure of GM differed markedly from that of healthy controls. Specifically, the relative abundance of potential pathogenic bacteria such as Bacteroides, Escherichia-Shigella, and Streptococcus within the γ-Proteobacteria phylum was increased in HSP patients, while the relative abundance of beneficial strains such as Prevotella_9 was decreased. These compositional changes in the gut microbiota may be closely related to the inflammatory processes of HSP (29). Furthermore, the abundance of Bacteroides is positively correlated with serum IgG levels in children with HSP, whereas the abundance of Lachnospiraceae is negatively correlated with complement component C3. Intake of Lactobacillus paracasei LC01 can reduce the abundance of Escherichia-Shigella in the gut, which is more abundant in recurrent HSP cases compared to initial cases and healthy controls. Although the study did not directly link Lactobacillus to C3, Lactobacillus may indirectly regulate immune response by reducing the abundance of Escherichia-Shigella, thereby affecting the complement system, particularly C3 levels. Escherichia-Shigella may play a role in promoting inflammation or immune dysregulation. Abdominal symptoms in HSP children were associated with specific gut microbiota, particularly Streptococcus and butyrate-producing bacteria. However, another study reported that the abundance of Bacteroides was positively correlated with serum IgG levels in patients, while the abundance of Lachnoclostridium was negatively correlated with complement component 3 (C3) levels (30). These correlations suggest that the gut microbiota may participate in the pathogenesis of HSP by influencing the host’s immune response. However, most current studies are primarily observational and have not directly demonstrated the impact of gut microbiota dysbiosis on the progression of HSP. Additionally, it remains unproven whether HSP patients can benefit from treatments involving probiotics or antibiotics (31). A previous MR study on the association between GM and HSP identified six GMs related to HSP, which differs significantly from our findings. In our study, we utilized updated GM data, which we believe lends greater credibility to our results (32).

Based on our analysis, we identified that the genus Blautia plays a crucial role in the immune cell-mediated GM pathway affecting HSP. Blautia is a significant component of the human gut microbiota and is closely linked to host health and disease states (33, 34). Research indicates that Blautia bacteria are vital in regulating host metabolism, enhancing gut barrier function, and participating in immune modulation. Specifically, Blautia coccoides and Blautia wexlerae have been shown to maintain colonic mucus function by secreting short-chain fatty acids (SCFAs) under low-fiber diets, which is essential for protecting against gut microbial invasion and maintaining intestinal health (35). In studies on childhood obesity, a reduction in Blautia species is associated with increased gut inflammation and worsening metabolic phenotypes. Specifically, decreased levels of Blautia luti and Blautia wexlerae in the gut of obese children are linked to the development of insulin resistance, potentially leading to more severe metabolic disorders such as type 2 diabetes (T2D). These findings underscore the potential role of Blautia bacteria in maintaining gut immune homeostasis and preventing obesity-related complications (36). Additionally, Blautia coccoides stimulates mucus growth by activating the short-chain fatty acid receptor Ffar2. This mechanism might provide a new target for restoring mucus growth in mucus-related disease states. Supplementation with Blautia coccoides under a low-fiber diet significantly increases the growth rate of colonic mucus in mice without altering its thickness. This suggests that Blautia bacteria may directly impact the integrity and function of the intestinal mucus layer through their metabolic products (34). Numerous studies have linked Blautia to various diseases, such as lower respiratory tract infections and colorectal cancer (37). However, we have not yet found research exploring the relationship between HSP and Blautia.

One of the pathological features of HSP is the deposition of IgA-containing immune complexes on the walls of small blood vessels. This process activates the complement system, triggering an inflammatory response (38). Abnormal polymeric forms of IgA are particularly prominent in HSP patients and may be associated with mucosal immune abnormalities. Furthermore, follicular helper T cells (Tfh) play a critical role in mucosal immunity by promoting IgA secretion through IL-21 production and direct interaction with B cells. Both cellular and humoral immunity are involved in the pathogenesis of HSP. Compared to healthy controls, HSP patients exhibit decreased levels of T lymphocyte subsets, B cells, and NK cells, while levels of IgG, IgA, IgM, and C3 are elevated, indicating an activated immune system (39). The abnormal elevation of Tfh cells is associated with increased IgA production, suggesting these cells play a key role in the development of HSP (38). Additionally, the immune cell subsets in HSP patients are altered, notably with reduced proportions of CD3+ and CD4+ cells, leading to a significant decrease in the CD4+/CD8+ ratio (40). This imbalance in T cell subsets may be related to the inflammatory process in HSP. Concurrently, the increased proportion of B cells and elevated immunoglobulin levels reflect the activation of humoral immunity. Renal involvement is a significant complication in HSP patients, with immunoglobulin and complement system activation playing a crucial role in kidney damage. The number of red blood cells in the urine correlates positively with IgA levels and negatively with serum complement C4 levels, indicating that complement activation may play a key role in HSP-related renal damage. Regulatory T cells (Treg) and regulatory B cells (Breg) also play important roles in immune regulation in HSP. The frequency of Treg cells is higher during the acute phase of HSP than during the remission phase and in healthy controls, while the percentage of Breg cells in HSP patients is associated with renal damage (6). These findings suggest that Treg and Breg cells may play significant roles in the immunoregulation of HSP.

Tregs play a crucial role in maintaining immune homeostasis and preventing various diseases. Through their immunoregulatory functions, Tregs effectively suppress excessive immune responses, reduce chronic inflammation, and are particularly important in the context of obesity and metabolic diseases (41). In studies on pediatric SARS-CoV-2-related multisystem inflammatory syndrome (MIS-C), Treg cell dysfunction has been linked to disease development. The discovery of the Notch1/CD22 signaling axis offers new insights into the role of Treg cells in MIS-C (42). Additionally, Treg cells suppress effector T cell activity and reduce inflammatory responses by secreting anti-inflammatory cytokines such as IL-10 and TGF-β. This function is particularly important in allergic reactions and autoimmune diseases. The characteristics and functional attributes of Treg cells have also been studied as potential biomarkers for predicting the development of allergies in children (43). Monocytes are white blood cells in the circulating blood, constituting a significant proportion of peripheral blood leukocytes in children. Originating from the bone marrow, they migrate through the bloodstream to various parts of the body, where they differentiate into macrophages and dendritic cells. These differentiated cells play crucial roles in maintaining tissue homeostasis and immune surveillance. In children, the development and functional maturation of monocytes are vital for combating infections and managing inflammatory responses (44, 45). During disease progression, the role of monocytes is equally significant. For example, in pediatric MIS-C, monocyte activation and abnormal proliferation are closely associated with the severity of the disease. Studies indicate that increased monocyte heterogeneity is linked to cardiovascular complications in MIS-C, providing potential biomarkers for early identification and treatment (46). Additionally, in other diseases such as autoimmune disorders, chronic infections, and tumors, monocytes play a critical role by secreting cytokines and chemokines. These secretions help regulate the inflammatory process and influence the behavior of other immune cells (47).

In this study, we employed MR analysis, an epidemiological technique that uses genetic variations as instrumental variables to explore potential causal relationships between GM, immune cells, and HSP. The advantage of MR analysis lies in its ability to provide more reliable causal inferences compared to traditional observational studies. By using genetic variations to mimic random allocation, MR analysis reduces the influence of confounding factors and selection bias. Additionally, MR analysis is not affected by reverse causation, which is particularly important when studying chronic diseases and complex biological pathways.

### Limitation

Despite certain advancements, this study has some limitations that suggest directions for future research. First, most study samples are primarily derived from populations of European ancestry, limiting the generalizability of the findings. Therefore, further studies are needed across different races and populations to validate the universality of the current findings. Second, due to the limited number of available genetic variants, some studies had to relax the significance threshold, which might affect the statistical power. Additionally, although the MR analysis design reduces the impact of confounding factors, it cannot completely eliminate all potential confounders, such as environmental factors and lifestyle. The validity of genetic instruments, issues of pleiotropy, biases in data sources, and the choice of statistical methods could also influence the study results. MR studies generally provide evidence of causality rather than directly investigating biological mechanisms, which requires further biological research to elucidate. Given that interpreting MR study results requires consideration within specific biological and epidemiological contexts, future research should be conducted within a broader scientific framework to ensure the robustness and generalizability of the results. Despite these limitations, our study offers valuable insights for further research on the relationships between GM, immune cells, and HSP.

## Conclusion

Through MR analysis, we revealed the potential causal relationships between GM, circulating immune cells immune cells, and HSP. We identified relevant pathogenic and probiotic bacterial groups and attempted to identify circulating immune cells that may act as mediators in these relationships. This study may aid in the early detection of HSP and provide new directions for prevention and treatment. Despite the limitations regarding sample selection and the representativeness of genetic variations, our comprehensive analysis offers new perspectives for research in this field and lays the groundwork for future studies.

## Data Availability

The original contributions presented in the study are included in the article/**Supplementary Material**. Further inquiries can be directed to the corresponding author.
