# Supplementary material for: Causal relationships between gut microbiota, immune cell, and Henoch-Schönlein Purpura: a two-step, two-sample Mendelian randomization study
Source: Front Immunol. 2024 Aug 14;15:1450544. doi: 10.3389/fimmu.2024.1450544 (PMC11349531; doi:10.3389/fimmu.2024.1450544)
Supplement: Supplementary file 2 [file Table2.docx]

**STROBE-MR checklist of recommended items to address in reports of Mendelian randomization studies**^1^ ^2^

| **Item No.** | **Section** | **Checklist item** | **Page No.** | **Relevant text from manuscript** |
| --- | --- | --- | --- | --- |
| 1 | **TITLE and ABSTRACT** | Indicate Mendelian randomization (MR) as the study’s design in the title and/or the abstract if that is a main purpose of the study |  | Title: Causal relationships between gut microbiota, immune cell, and Henoch-Schönlein Purpura: a two-step,two-sample Mendelian randomization study  Abstract:  Methods: We performed a two-sample Mendelian randomization study using an inverse variance weighted (IVW) method to examine the causal role of GM on HSP and the mediation effect of immune cells between the association of GM and HSP. |
|  | **INTRODUCTION** |  |  |  |
| 2 | **Background** | Explain the scientific background and rationale for the reported study. What is the exposure? Is a potential causal relationship between exposure and outcome plausible? Justify why MR is a helpful method to address the study question |  | Introduction:  The first six paragraphs of the Introduction section |
| 3 | **Objectives** | State specific objectives clearly, including pre-specified causal hypotheses (if any). State that MR is a method that, under specific assumptions, intends to estimate causal effects |  | Introduction:  In this study, we employed a two-step MR approach to determine: (i) whether gut microbiota is causally associated with HSP; (ii) whether immune cells are causally associated with HSP; and (iii) to assess the extent to which immune cells mediate the effects of gut microbiota on HSP. |
|  | **METHODS** |  |  |  |
| 4 | **Study design and data sources** | Present key elements of the study design early in the article. Consider including a table listing sources of data for all phases of the study. For each data source contributing to the analysis, describe the following: |  | Table listing sources of data for all phases of the study: Supplementary Table S1 |
|  | a) | Setting: Describe the study design and the underlying population, if possible. Describe the setting, locations, and relevant dates, including periods of recruitment, exposure, follow-up, and data collection, when available. |  | GWAS summary data sources  Microbiome Project provides gut microbiome data from a subset of 8,208 volunteers from the Dutch Lifeline population cohort.  The initial GWAS was conducted using data from 3,757 European individuals, with no overlapping cohorts, identifying approximately 17.6 million genetic variants. |
|  | b) | Participants: Give the eligibility criteria, and the sources and methods of selection of participants. Report the sample size, and whether any power or sample size calculations were carried out prior to the main analysis |  | GWAS summary data sources  GWAS summary statistics for each immune signature are publicly available from the GWAS Catalog (accession numbers GCST90001391 to GCST90002121)(Orrù et al., 2020). These data encompass 731 immunophenotypes |
|  | c) | Describe measurement, quality control and selection of genetic variants |  | Instrumental variable selection and data harmonization  we set the filtering conditions for SNPs to act as IVs for GMs and immune cell traits at a p-value threshold of less than 1e-5, However, when HSP was considered as the exposure, we applied an even stricter standard (p-value less than5e-6); we utilized the two-sample MR R package for cluster analysis, setting a threshold of R2 < 0.001 and a cluster distance of 10,000 kb(Slatkin, 2008, Wang et al., 2024). |
|  | d) | For each exposure, outcome, and other relevant variables, describe methods of assessment and diagnostic criteria for diseases |  | GWAS summary data sources  The definitions of exposure and outcome are detailed in the original article. |
|  | e) | Provide details of ethics committee approval and participant informed consent, if relevant |  | GWAS summary data sources  Genome-wide association studies aggregate statistical data on HSP, microbiota, and immune cells for MR analysis. Every GWAS participating in this research was disclosed via the initial study and obtained ethical clearance from their individual institutions. |
| 5 | **Assumptions** | Explicitly state the three core IV assumptions for the main analysis (relevance, independence and exclusion restriction) as well assumptions for any additional or sensitivity analysis |  | Study design  The MR analysis follows three fundamental principles: (I) the genetic variation is directly related to the exposure; (II) the genetic variation is not related to potential confounding factors between the exposure and the outcome; and (III) the genetic variation does not affect the outcome through pathways other than the exposure(Emdin et al., 2017). |
| 6 | **Statistical methods: main analysis** | Describe statistical methods and statistics used |  |  |
|  | a) | Describe how quantitative variables were handled in the analyses (i.e., scale, units, model) |  | Statistical analysis  Five MR analysis methods were employed: MR Egger, Weighted median, Inverse variance weighted (IVW), Simple mode, and Weighted mode. Among these, the IVW method was considered the primary approach for assessing causality due to its precision and robustness. |
|  | b) | Describe how genetic variants were handled in the analyses and, if applicable, how their weights were selected |  | Instrumental variable selection and data harmonization  we set the filtering conditions for SNPs to act as IVs for GMs and immune cell traits at a p-value threshold of less than 1e-5, However, when HSP was considered as the exposure, we applied an even stricter standard (p-value less than5e-6) |
|  | c) | Describe the MR estimator (e.g. two-stage least squares, Wald ratio) and related statistics. Detail the included covariates and, in case of two-sample MR, whether the same covariate set was used for adjustment in the two samples |  | Statistical analysis  Five MR analysis methods were employed: MR Egger, Weighted median, Inverse variance weighted (IVW), Simple mode, and Weighted mode. Among these, the IVW method was considered the primary approach for assessing causality due to its precision and robustness. Statistical significance was determined with a p-value threshold of less than 0.05, and the odds ratio (OR) was used to measure the association between exposure factors and outcomes. An OR greater than 1 indicated a positive association, while an OR less than 1 indicated a negative association(Zhu et al., 2022, Liu et al., 2022). |
|  | d) | Explain how missing data were addressed |  | GWAS summary data sources  Using a Sardinian sequence-based reference panel, around 22 million SNPs were imputed from high-density array genotyping. The associations were assessed while considering covariates, including sex, age, and age squared(Orrù et al., 2020).All data for GWAS is sourced from different alliances or agencies, so there is no duplication between samples. The definitions of exposure and outcome are detailed in the original article(Supplementary Table S1). |
|  | e) | If applicable, indicate how multiple testing was addressed |  | Statistical analysis  The false discovery rate (FDR) method was used for correction, ensuring that only results with p-values less than the FDR threshold were included(Newson, 2011) |
| 7 | **Assessment of assumptions** | Describe any methods or prior knowledge used to assess the assumptions or justify their validity |  | Instrumental variable selection and data harmonization  To mitigate biases arising from weak instrumental variables, we specifically calculated the F-statistic, with IVs having an F-statistic exceeding 10 deemed to possess adequate statistical strength. |
| 8 | **Sensitivity analyses and additional analyses** | Describe any sensitivity analyses or additional analyses performed (e.g. comparison of effect estimates from different approaches, independent replication, bias analytic techniques, validation of instruments, simulations) |  | Statistical analysis  During the analysis, Cochran's Q statistic was used to assess heterogeneity. In cases of significant heterogeneity, MR-Egger regression was applied to analyze potential pleiotropy. Pleiotropy was further assessed using the MR-Egger intercept test and the MR-PRESSO method. Leave-one-out sensitivity analysis was performed to evaluate the impact of individual SNPs on the overall causal effect. Additionally, funnel plots and scatter plots were employed to visually present potential pleiotropy(Burgess and Thompson, 2017). Given the multiple datasets processed and compared simultaneously, there was a risk of false-positive results due to random effects. Therefore, the false discovery rate (FDR) method was used for correction, ensuring that only results with p-values less than the FDR threshold were included(Newson, 2011). This comprehensive approach aimed to minimize bias and provide reliable estimates of causal relationships between exposures and outcomes. |
| 9 | **Software and pre-registration** |  |  |  |
|  | a) | Name statistical software and package(s), including version and settings used |  | Statistical analysis  This study utilized R statistical software version 4.3.2 and the packages "TwoSampleMR," "VariantAnnotation," and "ieugwasr" for two-sample MR analysis, aiming to explore the causal relationships between specific exposure factors and outcomes(Skrivankova et al., 2021) |
|  | b) | State whether the study protocol and details were pre-registered (as well as when and where) |  | This study protocol and details were not pre-registered. |
|  | **RESULTS** |  |  |  |
| 10 | **Descriptive data** |  |  |  |
|  | a) | Report the numbers of individuals at each stage of included studies and reasons for exclusion. Consider use of a flow diagram |  | GWAS summary data sources section |
|  | b) | Report summary statistics for phenotypic exposure(s), outcome(s), and other relevant variables (e.g. means, SDs, proportions) |  | Supplementary Table S1 |
|  | c) | If the data sources include meta-analyses of previous studies, provide the assessments of heterogeneity across these studies |  | Supplementary Table S2 |
|  | d) | For two-sample MR:  i.  Provide justification of the similarity of the genetic variant-exposure associations between the exposure and outcome samples  ii.  Provide information on the number of individuals who overlap between the exposure and outcome studies |  | GWAS summary data sources  All data for GWAS is sourced from different alliances or agencies, so there is no duplication between samples. |
| 11 | **Main results** |  |  |  |
|  | a) | Report the associations between genetic variant and exposure, and between genetic variant and outcome, preferably on an interpretable scale |  | Supplementary Table S2 |
|  | b) | Report MR estimates of the relationship between exposure and outcome, and the measures of uncertainty from the MR analysis, on an interpretable scale, such as odds ratio or relative risk per SD difference |  | Supplementary Table S3, S4 |
|  | c) | If relevant, consider translating estimates of relative risk into absolute risk for a meaningful time period |  | Table 1 |
|  | d) | Consider plots to visualize results (e.g. forest plot, scatterplot of associations between genetic variants and outcome versus between genetic variants and exposure) |  | Figure2, 3 ,4 |
| 12 | **Assessment of assumptions** |  |  |  |
|  | a) | Report the assessment of the validity of the assumptions |  | Supplementary Table S5 |
|  | b) | Report any additional statistics (e.g., assessments of heterogeneity across genetic variants, such as *I^2^*, Q statistic or E-value) |  | Supplementary Table S2 |
| 13 | **Sensitivity analyses and additional analyses** |  |  |  |
|  | a) | Report any sensitivity analyses to assess the robustness of the main results to violations of the assumptions |  | Results  Cochran's Q statistic, MR-Egger intercept test, and MR-PRESSO indicated no heterogeneity or horizontal pleiotropy in this MR analysis.  In this MR study, the p-values obtained from Cochran's Q test were greater than 0.05, indicating no significant heterogeneity. Additionally, both the MR-Egger intercept test and the MR-PRESSO analysis provided no evidence of horizontal pleiotropy (Supplementary Table S5). |
|  | b) | Report results from other sensitivity analyses or additional analyses |  | Results  Cochran's Q statistic, MR-Egger intercept test, and MR-PRESSO indicated no heterogeneity or horizontal pleiotropy in this MR analysis.  In this MR study, the p-values obtained from Cochran's Q test were greater than 0.05, indicating no significant heterogeneity. Additionally, both the MR-Egger intercept test and the MR-PRESSO analysis provided no evidence of horizontal pleiotropy (Supplementary Table S5). |
|  | c) | Report any assessment of direction of causal relationship (e.g., bidirectional MR) |  | Total effect of GM on HSP  As for the picked GMr, we conducted a reverse MR and did not detect any significant causal relationship among them. |
|  | d) | When relevant, report and compare with estimates from non-MR analyses |  | Discussion  However, we have not yet found research exploring the relationship between HSP and Blautia. |
|  | e) | Consider additional plots to visualize results (e.g., leave-one-out analyses) |  | Results  Furthermore, in the leave-one-out sensitivity analysis, no single SNP significantly violated the overall effect of GM on HSP. |
|  | **DISCUSSION** |  |  |  |
| 14 | **Key results** | Summarize key results with reference to study objectives |  | Discussion  In this study, we employed MR analysis, an epidemiological technique that uses genetic variations as instrumental variables to explore potential causal relationships between GM, immune cells, and HSP. The advantage of MR analysis lies in its ability to provide more reliable causal inferences compared to traditional observational studies. By using genetic variations to mimic random allocation, MR analysis reduces the influence of confounding factors and selection bias. Additionally, MR analysis is not affected by reverse causation, which is particularly important when studying chronic diseases and complex biological pathways. |
| 15 | **Limitations** | Discuss limitations of the study, taking into account the validity of the IV assumptions, other sources of potential bias, and imprecision. Discuss both direction and magnitude of any potential bias and any efforts to address them |  | limitation  Despite certain advancements, this study has some limitations that suggest directions for future research. First, most study samples are primarily derived from populations of European ancestry, limiting the generalizability of the findings. Therefore, further studies are needed across different races and populations to validate the universality of the current findings. Second, due to the limited number of available genetic variants, some studies had to relax the significance threshold, which might affect the statistical power. Additionally, although the MR analysis design reduces the impact of confounding factors, it cannot completely eliminate all potential confounders, such as environmental factors and lifestyle. The validity of genetic instruments, issues of pleiotropy, biases in data sources, and the choice of statistical methods could also influence the study results. MR studies generally provide evidence of causality rather than directly investigating biological mechanisms, which requires further biological research to elucidate. Given that interpreting MR study results requires consideration within specific biological and epidemiological contexts, future research should be conducted within a broader scientific framework to ensure the robustness and generalizability of the results. Despite these limitations, our study offers valuable insights for further research on the relationships between GM, immune cells, and HSP. |
| 16 | **Interpretation** |  |  |  |
|  | a) | Meaning: Give a cautious overall interpretation of results in the context of their limitations and in comparison with other studies |  | The discussion section of this article devotes the most space to discussing the content of this article, and reasonably explains the MR results by comparing them with several published studies. |
|  | b) | Mechanism: Discuss underlying biological mechanisms that could drive a potential causal relationship between the investigated exposure and the outcome, and whether the gene-environment equivalence assumption is reasonable. Use causal language carefully, clarifying that IV estimates may provide causal effects only under certain assumptions |  | Discussion  GM dysregulation may be related to the pathogenesis and clinical manifestations of HSP. An observational study analyzing 18 primary cases, 16 recurrent cases, and 23 healthy children found that the diversity and richness of GM in HSP patients were significantly reduced, and the structure of GM differed markedly from that of healthy controls. Specifically, the relative abundance of potential pathogenic bacteria such as Bacteroides, Escherichia-Shigella, and Streptococcus within the γ-Proteobacteria phylum was increased in HSP patients, while the relative abundance of beneficial strains such as Prevotella_9 was decreased. These compositional changes in the gut microbiota may be closely related to the inflammatory processes of HSP(Zhang et al., 2021). Furthermore, the abundance of Bacteroides is positively correlated with serum IgG levels in children with HSP, whereas the abundance of Lachnospiraceae is negatively correlated with complement component C3. Intake of Lactobacillus paracasei LC01 can reduce the abundance of Escherichia-Shigella in the gut, which is more abundant in recurrent HSP cases compared to initial cases and healthy controls. Although the study did not directly link Lactobacillus to C3, Lactobacillus may indirectly regulate immune response by reducing the abundance of Escherichia-Shigella, thereby affecting the complement system, particularly C3 levels. |
|  | c) | Clinical relevance: Discuss whether the results have clinical or public policy relevance, and to what extent they inform effect sizes of possible interventions |  | Conclusion  Through MR analysis, we revealed the potential causal relationships between GM, circulating immune cells immune cells, and HSP. We identified relevant pathogenic and probiotic bacterial groups and attempted to identify circulating immune cells that may act as mediators in these relationships. This study may aid in the early detection of HSP and provide new directions for prevention and treatment. Despite the limitations regarding sample selection and the representativeness of genetic variations, our comprehensive analysis offers new perspectives for research in this field and lays the groundwork for future studies. |
| 17 | **Generalizability** | Discuss the generalizability of the study results (a) to other populations, (b) across other exposure periods/timings, and (c) across other levels of exposure |  | Discussion  In this study, we employed MR analysis, an epidemiological technique that uses genetic variations as instrumental variables to explore potential causal relationships between GM, immune cells, and HSP. The advantage of MR analysis lies in its ability to provide more reliable causal inferences compared to traditional observational studies. By using genetic variations to mimic random allocation, MR analysis reduces the influence of confounding factors and selection bias. Additionally, MR analysis is not affected by reverse causation, which is particularly important when studying chronic diseases and complex biological pathways.  Conclusion  Through MR analysis, we revealed the potential causal relationships between GM, circulating immune cells immune cells, and HSP. We identified relevant pathogenic and probiotic bacterial groups and attempted to identify circulating immune cells that may act as mediators in these relationships. This study may aid in the early detection of HSP and provide new directions for prevention and treatment. Despite the limitations regarding sample selection and the representativeness of genetic variations, our comprehensive analysis offers new perspectives for research in this field and lays the groundwork for future studies |
|  | **OTHER INFORMATION** |  |  |  |
| 18 | **Funding** | Describe sources of funding and the role of funders in the present study and, if applicable, sources of funding for the databases and original study or studies on which the present study is based |  | The author(s) declare that no financial support was received for the research, authorship, and/or publication of this article. |
| 19 | **Data and data sharing** | Provide the data used to perform all analyses or report where and how the data can be accessed, and reference these sources in the article. Provide the statistical code needed to reproduce the results in the article, or report whether the code is publicly accessible and if so, where |  | Publicly available datasets were analyzed in this study. |
| 20 | **Conflicts of Interest** | All authors should declare all potential conflicts of interest |  | The authors declare that the research was conducted in the absence of any commercial or financial relationships that could be construed as a potential conflict of interest. |

This checklist is copyrighted by the Equator Network under the Creative Commons Attribution 3.0 Unported (CC BY 3.0) license.

1. Skrivankova VW, Richmond RC, Woolf BAR, Yarmolinsky J, Davies NM, Swanson SA, et al. Strengthening the Reporting of Observational Studies in Epidemiology using Mendelian Randomization (STROBE-MR) Statement. JAMA. 2021;under review.

2. Skrivankova VW, Richmond RC, Woolf BAR, Davies NM, Swanson SA, VanderWeele TJ, et al. Strengthening the Reporting of Observational Studies in Epidemiology using Mendelian Randomisation (STROBE-MR): Explanation and Elaboration. BMJ. 2021;375:n2233.
